# Ligand recognition and G-protein coupling selectivity of cholecystokinin A receptor

**DOI:** 10.1038/s41589-021-00841-3

**Published:** 2021-09-23

**Authors:** Qiufeng Liu, Dehua Yang, Youwen Zhuang, Tristan I. Croll, Xiaoqing Cai, Antao Dai, Xinheng He, Jia Duan, Wanchao Yin, Chenyu Ye, Fulai Zhou, Beili Wu, Qiang Zhao, H. Eric Xu, Ming-Wei Wang, Yi Jiang

**Affiliations:** 1 The CAS Key Laboratory of Receptor Research, Shanghai Institute of Materia Medica, Chinese Academy of Sciences, Shanghai, China; 2 University of Chinese Academy of Sciences, Beijing, China; 3 The National Center for Drug Screening, Shanghai Institute of Materia Medica, Chinese Academy of Sciences, Shanghai, China; 4 Department of Haematology, Cambridge Institute for Medical Research, University of Cambridge, Cambridge, UK; 5 School of Pharmacy, Fudan University, Shanghai, China; 6 School of Life Science and Technology, ShanghaiTech University, Shanghai, China; 7 CAS Center for Excellence in Biomacromolecules, Chinese Academy of Sciences, Beijing, China; 8 State Key Laboratory of Drug Research, Shanghai Institute of Materia Medica, Chinese Academy of Sciences, Shanghai, China; 9 School of Basic Medical Sciences, Fudan University, Shanghai, China

## Abstract

Cholecystokinin A receptor (CCK_A_R) belongs to family A G-protein-coupled receptors and regulates nutrient homeostasis upon stimulation by cholecystokinin (CCK). It is an attractive drug target for gastrointestinal and metabolic diseases. One distinguishing feature of CCK_A_R is its ability to interact with a sulfated ligand and to couple with divergent G-protein subtypes, including G_s_, G_i_ and G_q_. However, the basis for G-protein coupling promiscuity and ligand recognition by CCK_A_R remains unknown. Here, we present three cryo-electron microscopy structures of sulfated CCK-8-activated CCK_A_R in complex with G_s_, G_i_ and G_q_ heterotrimers, respectively. CCK_A_R presents a similar conformation in the three structures, whereas conformational differences in the ‘wavy hook’ of the Gα subunits and ICL3 of the receptor serve as determinants in G-protein coupling selectivity. Our findings provide a framework for understanding G-protein coupling promiscuity by CCK_A_R and uncover the mechanism of receptor recognition by sulfated CCK-8.

Cholecystokinin (CCK), one of the earliest discovered gastrointestinal hormones, participates in gallbladder contraction and pancreatic enzyme secretion. It also acts as a neurotransmitter and is extensively distributed throughout the nervous system^1^. Selective cleavage of the CCK precursor produces a series of bioactive isoforms of different lengths, with CCK-58, CCK-33, CCK-22 and CCK-8 comprising the major peptide fragments in humans. However, the carboxy-terminal octapeptide CCK-8 (DYMGWMDF) is well conserved across species and is the smallest form that retains the full range of biological actions^2^, mediated by two CCK receptor subtypes (CCK_A_R and CCK_B_R), which are present throughout the central nervous system and the gut. CCK_A_R is primarily expressed in the alimentary tract, while CCK_B_R is mainly found in the brain and the stomach^3^. CCK_A_R has an ~500-fold higher affinity to CCK with a sulfated tyrosine, whereas CCK_B_R discriminates poorly between sulfated and non-sulfated CCK^4^.

CCK regulates appetite and food intake primarily through CCK_A_R on the vagal afferent neurons^5–8^, making CCK_A_R an attractive therapeutic target for obesity. However, the development of drugs against CCK_A_R is challenging, partly due to limited efficacy and safety concerns. Although several drug candidates are undergoing clinical trials, none has been approved so far^9,10^. Extensive efforts have been made to elucidate the mechanism of agonism at CCK_A_R through mutagenesis studies based on modeled receptor structures^11–15^, but the lack of precise structural information largely impedes our understanding of the molecular details of ligand recognition and receptor activation, and thus the drug discovery targeting CCK_A_R.

Most G-protein-coupled receptors (GPCRs) are known to couple with a specific subtype of G protein to elicit intracellular signal transduction^16–21^. There are four G-protein subtypes—stimulatory G protein (G_s_), inhibitory G proteins (G_i_) G_q_ and G_12/13_—participating in signaling pathways involving cyclic adenosine monophosphate (G_s_ and G_i_), calcium (G_q_) and small G protein (G_12/13_). A number of GPCR-G protein complex structures reported recently reveal that the primary determinants of G-protein coupling selectivity reside in the C-terminal α5-helix of the Gα subunit and relative outward movement of transmembrane helix 6 (TM6)^22,23^. However, CCK_A_R is different from most GPCRs as a result of its ability to couple with several G-protein subtypes. Activation of CCK_A_R elicits a diversified G-protein coupling pattern^24^, predominantly G_q_ (^25^), but G_s_ (^26^), G_i_ (^25,27^) and G_13_ (^28,29^) all play roles in CCK_A_R signaling. This unique feature makes CCK_A_R an ideal model to study G-protein selectivity and promiscuity (Fig. 1a). Here, we report three cryo-EM structures of sulfated CCK-8-activated CCK_A_R in complex with heterotrimeric G_q_, G_s_ or G_i_ protein, respectively. These structures reveal the unique binding mode in ligand recognition and the structural determinants responsible for the G-protein selectivity and promiscuity of CCK_A_R.

## Overall structures of CCK_A_R coupled to different G proteins

**Fig. 1:**
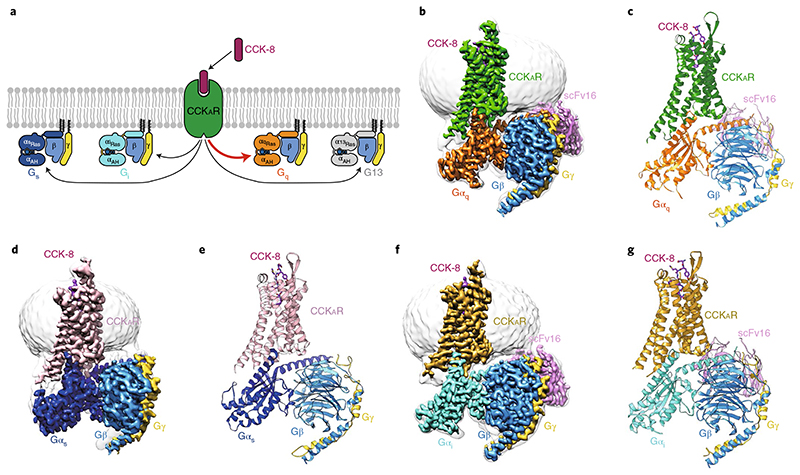
Cryo-EM structures of CCK_A_R–G protein complexes. **a**, Schematic of G-protein coupling promiscuity of CCK_A_R. **b**-**g**, Three-dimensional maps and models of the CCK-8–CCK_A_R–G_q_–scFv16 (**b**,**c**), CCK-8–CCK_A_R–G_s_ (**d**,**e**) and CCK-8–CCK_A_R–G_i_–scFv16 (**f**,**g**) complexes. CCK-8, magenta; G_q_-coupled CCK_A_R, green (**b**,**c**); G_s_-coupled CCK_A_R, pink (**d**,**e**); G_i_-coupled CCK_A_R, dark yellow (**f**,**g**); G**α**_q_, orange; G**α**_s_, blue; Gα_i_, cyan; G**β**, light blue; G**γ**, yellow; scFv16, light purple.

The structures of sulfated CCK-8-bound CCK_A_R in complex with G_q_, G_s_ or G_i_ heterotrimers were determined by single-particle cryo-EM at global resolutions of 2.9 Å, 3.1 Å and 3.2 Å, respectively (Fig. 1b-g, Supplementary Figs. 1–3 and Supplementary Table 1). Sulfated CCK-8 (DY^SO3H^MGMWDF-NH_2_), the highest-affinity natural ligand of CCK_A_R^4^, was used to assemble the CCK_A_R-G protein complexes. Three G-protein subtypes were engineered to stabilize the CCK_A_R–G protein complexes (Supplementary Fig. 4). Gα_q_ is chimerized by replacing its α*N*-helix with the equivalent region of Gα_i1_ to facilitate scFv16 binding^30^. Gα_s_ was modified based on the mini-Gα_s_ that was used in the crystal structure determination of the G_s_-coupled adenosine A_2A_ receptor (A_2A_R)^31^. Two dominant-negative (DN) mutations (G203A and A326S^32^) were introduced to Gα_i1_, and corresponding DN mutations at equivalent sites of Gα_s_ and Gα_q_ were also introduced (Supplementary Fig. 4b). Unless otherwise specified, G_q_, G_s_ and G_i_ refer to the respective engineered G proteins that are used in the CCK_A_R structure determination.

The final structures of the CCK-8–CCK_A_R–G protein complexes contain sulfated CCK-8 (residues D^1P^-F^8P^), Gα Ras-like domain, Gβγ subunits, scFv16 and the CCK_A_R residues (E38^N_term^-F385^8.58^, superscripts refer to Ballesteros–Weinstein numbering^33^). The majority of amino-acid side chains, including CCK-8, transmembrane domain (TMD), intracellular loops (ICLs 1–3) and extracellular loops (ECLs 1–3) were well resolved in the final models (Supplementary Fig. 5). Thus, the complex structures provide reliable details to study mechanisms of ligand recognition and G-protein coupling.

**Extended Data Fig. 1:**
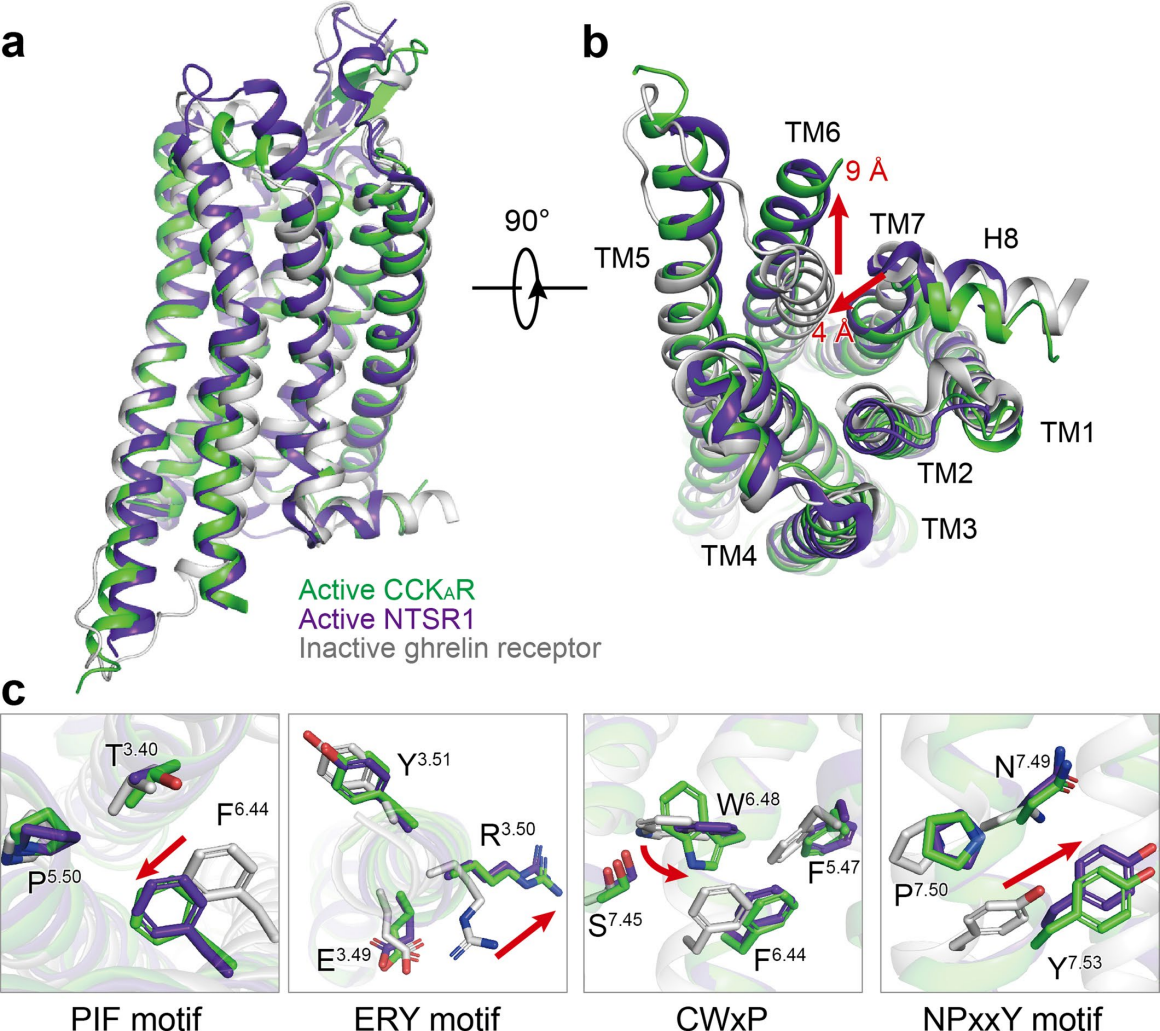
Active conformation of CCK_A_R. **a-b**, Structural comparison of inactive ghrelin receptor (grey), active NTSR1 (purple blue), and active CCK_A_R (green). Side view (**a**) and intracellular view (**b**) of the overall comparison are shown. **c**, Structural rearrangements of key activation motifs (PIF, ERY, CWxP, and NPxxY) in CCK_A_R compared to inactive ghrelin receptor and active NTSR1. NTSR1, neurotensin receptor 1.

Globally, CCK_A_R adopts similar overall conformations in all three structures, with an all-atom root-mean-square deviation (r.m.s.d.) of 0.84 for G_q_/G_s_-coupled receptors and 1.03 for G_q_/G_i_-coupled receptors. The structure of the CCK-8–CCK_A_R–G_q_ complex, which has the highest resolution at 2.9 Å, was used for detailed analysis and mechanistic evaluation of ligand recognition and receptor activation. The inactive and active structures of the closed homolog receptors (inactive: ghrelin receptor, PDB 6KO5 ^34^; active: neurotensin receptor 1 (NTSR1), PDB 6OS9 ^18^), all belonging to the β-branch of the rhodopsin family, are applied for structural comparison. CCK_A_R presents a fully active conformation, resembling the G_i_-coupled NTSR1, displaying an ~9-Å outward movement of TM6 (measured at the Cα of the residue at position 6.27 in CCK_A_R and the ghrelin receptor) and an ~4-Å inward shift of TM7 (Cα carbons of Y7.53) compared with the inactive ghrelin receptor (Extended Data Fig. 1a,b). Similar to the active NTSR1 complex, the conserved residues in the ‘micro-switches’ (PIF, ERY, CWxP and NPxxY) of CCK_A_R display the conserved conformations observed in active GPCRs (Extended Data Fig. 1c).

## Recognition of sulfated cholecystokinin

**Extended Data Fig. 2:**
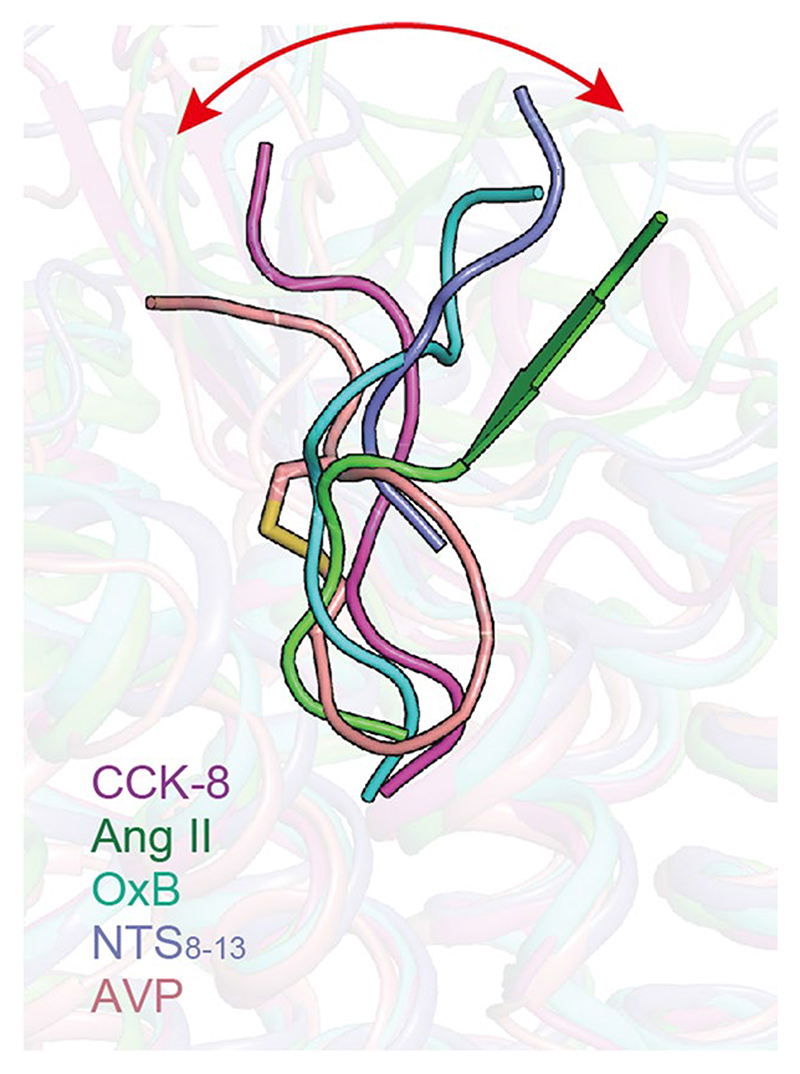
Structure comparison of CCK-8 with other neuropeptides solved to date. The neuropeptides are shown as a cartoon. The shift of the extracellular part of neuropeptides is highlighted as a red arrow. CCK-8 in the CCK-8–CCK_A_R–G_q_ complex structure, magenta; Ang II, angiotensin II (PDB: 6OS0), green; OxB, orexin B (PDB: 7L1U), cyan; NTS_8-13_, neurotensin 8-13 (PDB: 6OS9), purple blue; AVP, arginine vasopressin (PDB: 7DW9), salmon.

**Extended Data Fig. 3:**
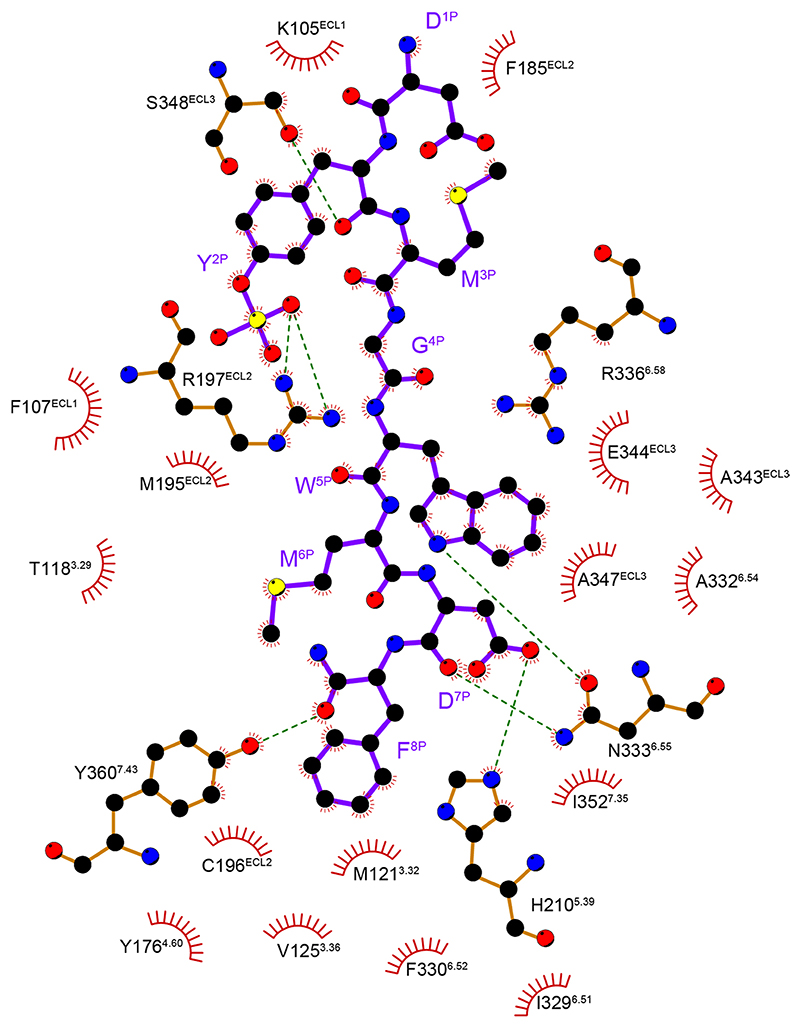
2D interaction plot of CCK_A_R recognition by sulfated CCK-8. Residues in the ligand-binding pocket are colored in green. CCK-8 is displayed as magenta sticks. Polar interactions are indicated as red dashed lines.

**Fig. 2:**
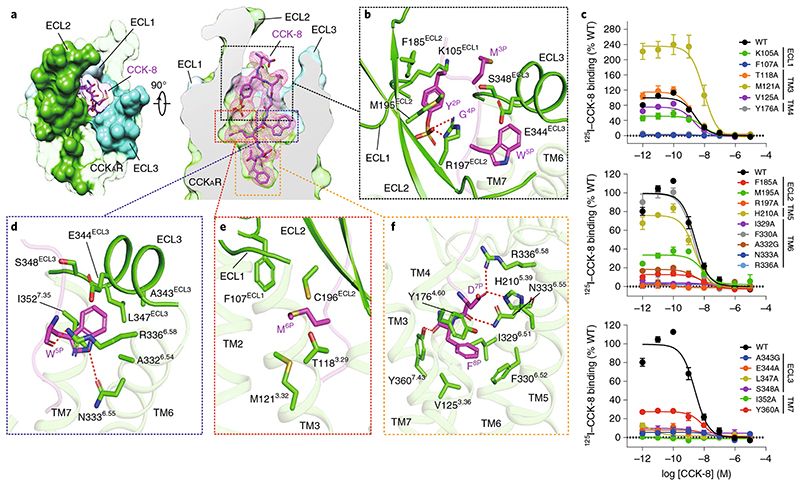
Recognition of sulfated CCK-8 by CCK_A_R. **a**, CCK-8 sits in the orthosteric binding pocket of CCK_A_R, as shown in an extracellular view (left) and side view (right). The density map of CCK-8 is shown as a magenta mesh, and CCK-8 is displayed as magenta sticks. CCK_A_R is shown in green as a cutaway surface (right). ECL1 (light blue), ECL2 (lime green) and ECL3 (turquoise) are highlighted as solid surfaces. **b**, Detailed interactions between sulfated CCK-8 and three extracellular loops of CCK_A_R. **c**, Effects of mutations in the receptor ligand-binding pocket on CCK-8 binding activity assessed by a radiolabeled ligand-binding assay. Data are presented as mean **±** s.e.m. of three independent experiments (*n* **=** 3), except for the WT (*n* **=** 4), and conducted in triplicate. Competition curves of mutants from ECL1, TM3 and TM4 (upper), ECL2, TM5 and TM6 (middle), and ECL3 and TM7 (bottom) compared to WT CCK_A_R are shown. **d**, Recognition of CCK-8 by the deep hydrophobic cavity beneath ECL3 of CCK_A_R. **e**, Recognition of CCK-8 by the shallow hydrophobic cavity beneath ECL1 and ECL2 of CCK_A_R. **f**, Recognition of CCK-8 by the bottom TMD region of CCK_A_R. Key interaction residues from CCK_A_R are shown as green sticks, and the receptor is shown in cartoon presentation. Polar interactions are indicated as red dashed lines.

Sulfated CCK-8 occupies the orthosteric binding pocket composed of TM3, TM4, TM5–7 and ECL1–3 (Fig. 2 and Extended Data Figs. 2 and 3), with its C terminus inserting into the TMD bundle and the N terminus facing the extracellular vestibule (Fig. 2a). The binding pocket of CCK-8 is largely overlapped with that of other reported endogenous neuropeptides, such as neurotensin (NTS_8-13_, PDB 6OS9 ^18^), angiotensin II (Ang II, PDB 6OS0 ^35^), orexin B (OXB, PDB 7L1U ^36^) and arginine vasopressin (AVP, PDB 7DW9 ^37^). It is noteworthy that the extracellular sides of these neuropeptides undergo remarkable conformational shifts, while their intracellular parts converge in an approximately overlapped position at the bottom of the binding pocket (Extended Data Fig. 2).

It is of interest that the octapeptide CCK-8 almost completely occupies the polypeptide-binding pocket, structurally supporting the fact that it is the smallest active form of CCK isoforms. The binding modes of CCK-8 are highly conserved in all three CCK_A_R–G protein complexes (all-atom r.m.s.d. of 0.71 for CCK-8 in G_q_/G_s_-coupled complexes, and 1.18 for CCK-8 in G_q_/G_i_-coupled complexes), supported by clear EM density maps (Fig. 2a and Supplementary Fig. 5). The region of ligand recognition by CCK_A_R can be divided into three major parts: (1) the extracellular loops, (2) hydrophobic cavities beneath ECLs and (3) the bottom of the TMD pocket (Fig. 2a).

**Extended Data Fig. 4:**
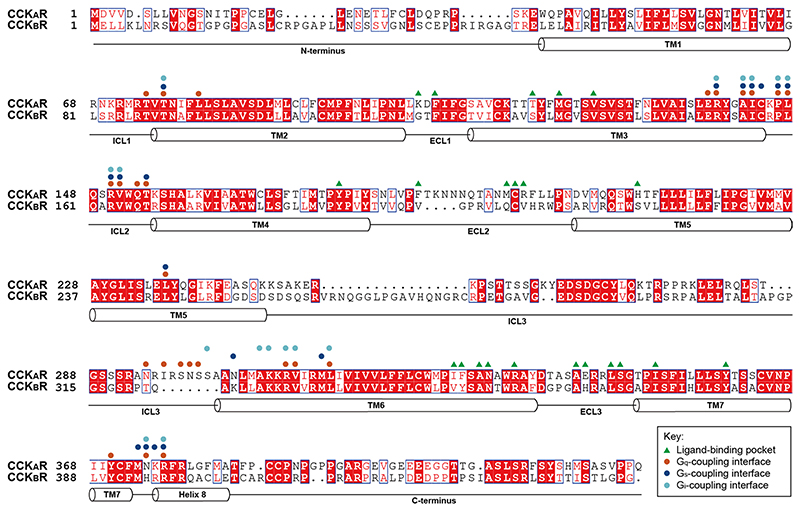
Sequence alignment of CCK receptors. Helical secondary structures are shown based on CCK_A_R. Residues involved in ligand-binding are labeled with green triangles. Residues involved in G protein coupling are labeled with circles (orange, G_q_; blue, G_s_; cyan, G_1_).

At the extracellular side, three ECLs are folded to embrace the N-terminal amino acids of CCK-8 (Fig. 2a). The sulfate group of ionic Y^2P^ interacts with the side chain of R197^ECL2^. This polar interaction prompts the aromatic ring of Y^2P^ to form hydrophobic contacts with F185^ECL2^, M195^ECL2^ and the main chain of K105^ECL1^, thus connecting CCK-8 to ECL1 and ECL2 (Fig. 2b and Extended Data Fig. 3). These structural observations are consistent with the previous finding that the R197^ECL2^M mutation was 1,470-fold less potent than the wild-type (WT) CCK_A_R^11^. The alanine mutation of R197^ECL2^ completely abolishes the binding of CCK-8, thus strongly supporting the contention that R197^ECL2^ serves as a determinant to discriminate between sulfated and non-sulfated CCK (Fig. 2c and Supplementary Table 2). Likewise, poor ligand selectivity of CCK_B_R may be attributed to a substitution of arginine for valine at the corresponding position (Extended Data Fig. 4). Meanwhile, M^3P^, G^4P^ and W^5P^ clamp the interior surface of ECL3 (Fig. 2b).

Two hydrophobic cavities exist below the ECLs to accommodate W^5P^ and M^6P^ (Fig. 2d,e). The side chain of W^5P^ is sandwiched by the side chains of I352^7.35^ and R336^6.58^ and buries in a deep hydrophobic pocket composed of TM6, ECL3 and TM7 (Fig. 2d). The backbone CO group of W^5P^ forms a hydrogen bond with R336^6.58^, and its indole nitrogen atom makes another hydrogen bond with N333^6.55^ (Fig. 2d and Extended Data Fig. 3), which is reported to be critical to CCK_A_R activation^38^. Alanine mutations in residues N333^6.55^, R336^6.58^, A343^ECL3^, E344^ECL3^, L347^ECL3^ and S348^ECL3^ completely abolish the binding of CCK-8, suggesting the key roles of these residues in CCK-8 recognition (Fig. 2c and Supplementary Table 2). In contrast to the W^5P^-occupied hydrophobic pocket, M^6P^ sits in a relatively shallow hydrophobic cavity in the opposite direction, constituted by F107^ECL1^, C196^ECL2^, T118^3.29^ and M121^3.32^ (Fig. 2e and Extended Data Fig. 3). Mutating F107^ECL1^ and residues in ECL2 and ECL3 to alanine eliminated the binding ability of CCK-8 entirely, highlighting an essential function of the three ECLs in peptide recognition (Fig. 2c and Supplementary Table 2).

At the bottom of the binding pocket, D^7P^ and main chain CO group of CCK-8 form a stabilizing polar interaction network with TM5 (H210^5.39^), TM6 (N333^6.55^ and R336^6.58^) and TM7 (Y360^7.43^) (Fig. 2f and Extended Data Fig. 3). The phenyl ring of F^8P^ makes a polar hydrogen-*π* interaction with Y176^4.60^, and inserts into a large hydrophobic crevice composed of residues from TM3, TM4, TM5 and TM6 (Fig. 2f and Extended Data Fig. 3). Besides N333^6.58^ and R336^6.58^, which also interact in a polar manner with W^5P^, I329^6.51^ is closely related to CCK-8 binding (Fig. 2c and Supplementary Table 2).

**Extended Data Fig. 5:**
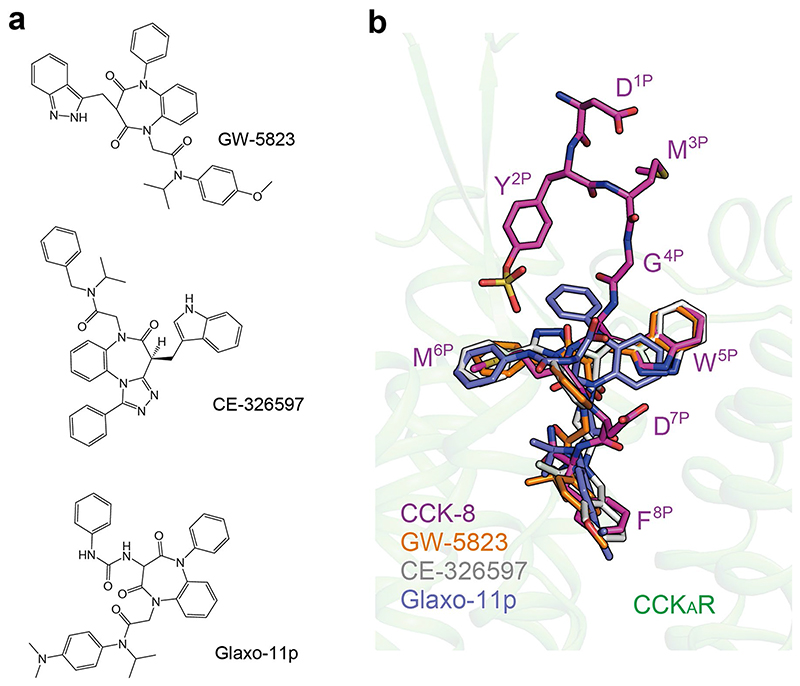
Molecular docking of small molecule agonists to the CCK_A_R structure. **a**, Chemical structures of small molecule agonists of CCK_A_R. **b**, Comparison of the binding poses of three agonists with CCK-8. CCK-8, magenta; GW-5823, orange; CE-326597, grey; Glaxo-11p, purple blue. CCK-8 and small molecule agonists are shown as sticks. The amino acids of CCK-8 are labelled.

Elucidation of the recognition mechanism of CCK-8 provides clues for therapeutic development against CCK_A_R. GW-5823, CE-326597 and Glaxo-11p (Extended Data Fig. 5a) are small-molecule agonists for CCK_A_R with moderate activities^10,39,40^. Docking of these agonists to the CCK_A_R shows that they only occupy the bottom half of the TMD binding pocket, thus lacking essential interactions with ECLs 1–3 of CCK_A_R (Extended Data Fig. 5b). This structural feature may lead to a weaker activity of these small-molecule agonists relative to CCK-8. Together, our data provide a framework for understanding the mechanism of small-molecule agonist recognition and offer a template for guiding drug design targeting CCK_A_R.

## Overall coupling mode of CCK_A_R–G protein complexes

**Extended Data Fig. 6:**
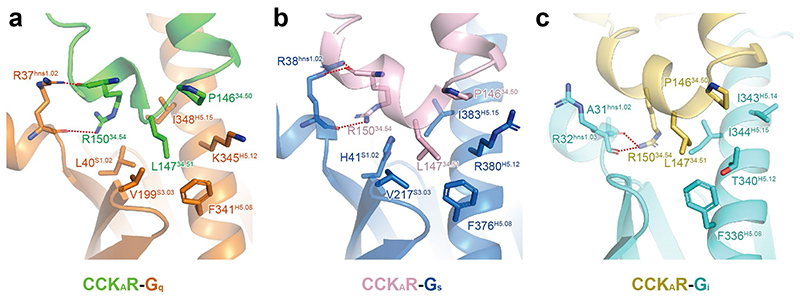
The interface between CCK_A_R ICL2 and different G proteins. Detailed interaction between the receptor and G**α**_q_ (**a**), G**α**_s_ (**b**), and G**α**_i_ (**c**) are shown. Side chains of related residues are shown as sticks.

**Fig. 3:**
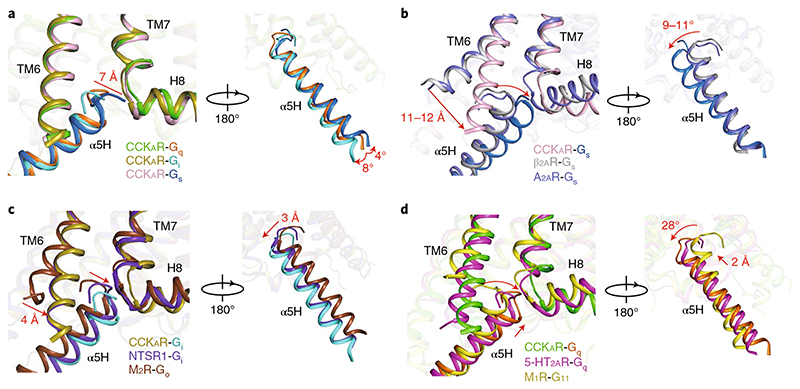
Structural comparison of TM6 and the α5-helix between CCK_A_R–G protein complexes and representative G_s_-, G_q_- and G_i_-coupled GPCR structures, in two different views. **a**, Structural comparison of CCK_A_R–G_q_, CCK_A_R–G_s_ and CCK_A_R–G_i_ complexes. A 7-Å movement of the distal end of the G**α**_s_ **α**5-helix (**α**5H) relative to that of G**α**_q_ and a swing of the G**α α**5-helix are highlighted as red arrows. **b**, Structural comparison of CCK_A_R–G_s_ with **β**_2A_R–G_s_ and A_2A_R–G_s_ complexes. Red arrows indicate an 11–12-Å displacement of TM6 and a 9–11° swing of the G**α α**5-helix of G_s_-coupled CCK_A_R relative to G_s_-coupled **β**_2A_R and A_2A_R. **c**, Structural comparison of CCK_A_R–G_i_ with the NTSR1–G_i_ and M_2_R–G_o_ complexes. A 4-Å inward displacement of TM6 and a 3-Å G**α_i_** **α**5-helix shift of G_q_-coupled CCK_A_R in comparison to G_o_-coupled M_2_R are indicated as red arrows. **d**, Structural comparison of CCK_A_R–G_q_ with 5-HT_2A_R–G_q_ and M_1_R–G_11_ complexes. A 2-Å upward movement of G**α**_q_ of G_q_-coupled CCK_A_R compared to G_q_-coupled 5-HT_2A_R and a 28° rotation relative to G_11_-coupled M_1_R are highlighted as red arrows. The complex structures are aligned based on TM2–TM4 of the receptors. **β**_2A_R–G_s_, A_2A_R–G_s_, NTSR1–G_i_, M_2_R–G_o_, 5-HT_2A_R–G_q_ and M_1_R–G_11_ structures (PDB 3SN6, 5G53, 6OS9, 6OIK, 6WHA and 6OIJ) are colored in gray, marine, purple blue, dark brown, magenta and yellow, respectively.

Although all four G-protein subtypes were reported to interact with CCK_A_R^24^, only three of the CCK_A_R–G protein samples (CCK_A_R–G_q_, CCK_A_R–G_s_ and CCK_A_R–G_i_ protein complexes) were obtained for high-resolution cryo-EM structure determination (Fig. 1). Structural comparison indicated that TM6 and ICL2 in CCK_A_R adopt nearly identical conformations in G_q_-, G_i_- and G_s_-coupled structures (Fig. 3a and Extended Data Fig. 6a–c). However, slightly different tilts of the Gα α5-helix were seen among the three heterotrimeric G proteins (4° for Gα_q_/Gα_s_ and 8° for Gα_q_/Gα_i_; Fig. 3a). Meanwhile, the distal end of the Gα_s_ α5-helix moves 7 Å outward, away from the TMD core relative to the equivalent Gα_q_ residue (measured at the Cα atom of L^H5.25^, where superscripts refer to the common Gα numbering (CGN) system^41^; Fig. 3a). G_q_ presents the largest solvent-accessible surface area (SASA, 1,492 Å^2^) with the receptor, compared to a G_s_ value of 1,293 Å^2^ and G_i_ value of 1,167 Å^2^, consistent with a 6.6- to 20.3-fold increased potency of G_q_ coupling to CCK_A_R in comparison to coupling with G_s_ and G_i_ (Supplementary Table 3). This finding supports the hypothesis that the size of the G-protein coupling interface may correlate with the ability of a receptor to link with different G proteins^21,22^. In addition, coupling of different G-protein subtypes exhibits distinct effects on CCK-8 binding. Compared to G_s_ or G_i_ proteins, G_q_ coupling increases the binding affinity of CCK-8 (Supplementary Table 3), consistent with the increased binding activity of isoproterenol against β_2A_R in the presence of G_s_ protein^16^. This finding indicates an allosteric modulation effect of G_q_ protein on CCK-8 binding, supporting the positive cooperativity between agonists and G proteins^42^.

In addition, comparisons of these three complex structures to previously reported G-protein-coupled class A GPCRs reveal the different extent of TM6 displacement and the concomitant shift of the Gα α5-helix (Fig. 3b-d). TM6 of CCK_A_R in all three G-protein complexes displays an 11–12-Å (measured at the Cα atom of the residue at position 6.27) smaller outward displacement compared to G_s_-coupled GPCRs, which translates into a notable swing of the Gα α5-helix in the same direction (9–11° relative to G_s_-coupled β_2_AR and A_2A_R as measured at the Cα atom of Y^H5.23^). This smaller displacement of TM6 is contrary to the previous assumption that TM6 of G_s_-coupled GPCRs undergoes a notable outward movement, thus opening a larger cytoplasmic pocket to accommodate bulkier residues at the distal end of the Gα_s_ α5-helix relative to G_i/o_-coupled receptors^21,43^. To avoid a potential clash with TM6, the distal end of the Gα_s_ α5-helix in the CCK_A_R–G_s_ complex stretches away from the TMD core and inserts into the crevice between the TM6 and TM7–helix 8 joint. This featured conformation of the Gα_s_ α5-helix in the CCK_A_R–G_s_ complex is unique compared to that in structures of the G_s_-coupled β_2A_R and A_2A_R, supporting the complexity of the GPCR–G protein coupling mechanism (Fig. 3b).

TM6 and the Gα α5-helix of CCK_A_R–G protein complexes display similar conformational changes to other G_i_- and G_q_-coupled GPCRs, such as the G_i_-coupled NTSR1 and the G_q_-coupled 5-HT_2A_R (Fig. 3c,d). TM6 of the CCKA_R_–G_i_ protein complex is highly overlaid with that of G_i_-coupled NTSR1, while the cytoplasmic end of TM6 shows a 4-Å smaller outward displacement compared to that of G_o_-coupled M_2_R (Fig. 3c). On the G-protein side, the α5-helix of G_αi_ in the CCK_A_R-G_i_ complex shows a nearly overlapped conformation with that of the NTSR1-G_i_ complex. In contrast, it exhibits a 3-Å (measured at the Cα atom of Y^H5.23^) shift away from TM6 relative to that of G_o_-coupled M_2_R (Fig. 3c). Structural comparison of G_q_-coupled CCK_A_R with G_q_/G_11_-coupled GPCRs demonstrates a 2-Å (measured at the Cα atom of Y^H5.23^) upward shift toward the cytoplasmic cavity in comparison to the G_q_-coupled 5-HT_2A_R and a 28° rotation away from TM6 relative to G_11_-coupled M_1_R (Fig. 3d).

## Interactions of the ‘wavy hook’ of CCK_A_R-G protein complexes

The ‘wavy hook’ at the extreme C terminus of the Gα α5-helix is thought to be one of the coupling specificity determinants for G proteins^44,45^, and undergoes distinct conformational rearrangements among the three CCK_A_R-G protein complexes (Fig. 3a).

**Fig. 4:**
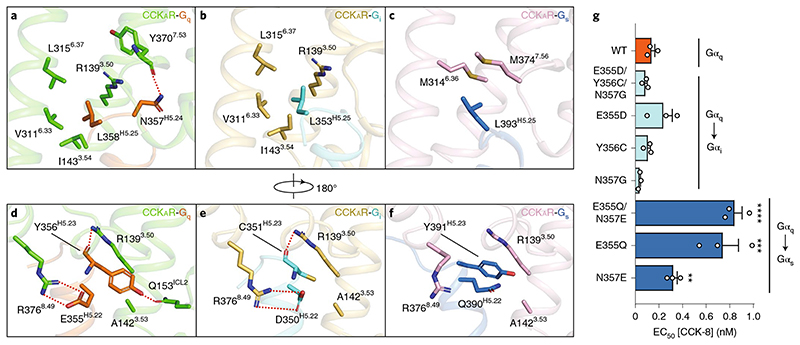
Distinct interaction patterns of residues from the ‘wavy hook’ motif. **a**–**c**, Details of the interaction between CCK_A_R and L358^H5.25^ and N357^H5.24^ of the G**α**_q_ subunit (**a**), L353^H5.25^ of the Gα_i_ subunit (**b**) and L393^H5.25^ of the G**α**_s_ subunit (**c**). **d**–**f**, Details of the interaction between CCK_A_R and Y356^H5.23^ and E355^H5.22^ of the G**α**_q_ subunit (**d**), C351^H5.23^ and D350^H5.22^ of the G**α**_i_ subunit (**e**) and Y391^H5.23^ and Q390^H5.22^ of the G**α**_s_ subunit (**f**). Hydrogen bonds and salt bridges are indicated as red dashed lines. **g**, BRET assay evaluating the effects of ‘wavy hook’ substitutions on CCK_A_R-G protein coupling. The ‘wavy hook’ residues of the G**α**_q_ subunit were displaced by the corresponding residues in the G**α**_s_ and G**α**_i_ subunits. Data are shown as mean **±** s.e.m. of three independent experiments (*n* **=** 3), conducted in triplicate. All data were analyzed by one-way analysis of variance Dunnett multiple comparisons test (*P* **=** 1, *P =* 0.9827, *P* **=** 0.7521, *P* **=** 0.9994, *P* **=** 0.7421, *P* < 0.0001, *P* < 0.0001 and *P* **=** 0.2078 from top to bottom, ***P < 0.001, ****P < 0.0001 versus WT).

A structural comparison of the interaction interface between the receptor cytoplasmic cavity and the Gα ‘wavy hook’ reveals distinct features of CCK_A_R-G protein coupling. Well-defined densities of Gα-protein ‘wavy hook’ residues allow for detailed structural analyses except for residues at the -1 position. The L(-2)^H5.25^ in the α5-helix is highly conserved across the G-protein families and plays a pivotal role in G-protein coupling. Both L358^H5.25^ in Gα_q_ and L353^H5.25^ in Gα_i_ hydrophobically interact with residues in TM3 and TM6 (R139^3.50^, I143^3.54^, V311^6.33^ and L315^6.37^; Fig. 4a,b). Owing to the notable displacement of the Gα_s_ C terminus, L393^H5.25^ in Gα_s_ moves 7 Å outward away from the TMD core relative to the equivalent Gα_q_ residue (Fig. 3a), repositioning it in a hydrophobic subpocket formed by M314^6.36^ and M373^7.56^ (Fig. 4c). In contrast to L(-2)^H5.25^, residues at positions H(-3)^5.24^, H(-4)^5.23^ and H(-5) ^5.22^ are less conserved. N357(-3)^H5.24^ in Gα_q_ makes a hydrogen bond with the backbone CO group of Y370^7.53^ (Fig. 4a). As a result of the replacement of Gα_i_ G352(-3)^H5.24^ and the repositioning of Gα_s_ E392(-3)^H5.24^, the corresponding hydrogen bond is absent in CCK_A_R-G_i_ and CCK_A_R-G_s_ complex structures. Additionally, Y356(-4)^H5.23^ in Gα_q_ forms extensive interactions with the receptor cytoplasmic cavity by making hydrogen bonds with R139^3.50^ and Q153^ICL2^ (Fig. 4d). By contrast, C351(-4)^H5.23^ in Gα_i_ only forms a weak hydrogen bond with R139^3.50^ via its backbone CO group (Fig. 4e). Y391(-4)^H5.23^ in Gα_s_ exhibits limited hydrophobic and van der Waals interactions with residues in TM2 and TM3 (T76^2.39^, R139^3.50^ and A142^3.53^; Fig. 4f). Furthermore, both E355(-5)^H5.22^ in Gα_q_ and D350(-5)^H5.22^ in Gα_i_ form salt bridges with R376^8.49^ in CCK_A_R, while Q390(-5)^H5.22^ in Gα_s_ disfavors the formation of the corresponding electrostatic interaction (Fig. 4d-f). To understand the ‘wavy hook’-mediated G-protein selectivity, we displaced the amino acids (H5.22-H5.25) in the Gα_q_ subunit with the corresponding ones in the Gα_s_ and Gα_i_ subunits. Bioluminescence resonance energy transfer (BRET) assay results show that the Gα_i_ displacement has no impact on CCK_A_R-G protein coupling compared to the WT Gα_q_ subunit. However, partial (E355Q or N357E) or complete Gα_s_ substitution remarkably decreased the G-protein coupling activity of CCK_A_R (Fig. 4g). These results indicate that the ‘wavy hook’ may play a crucial role in the coupling selectivity of CCK_A_R with G_q_ over the G_s_ protein.

## Contribution of CCK_A_R ICL3 to G_q_ coupling selectivity

**Extended Data Fig. 7:**
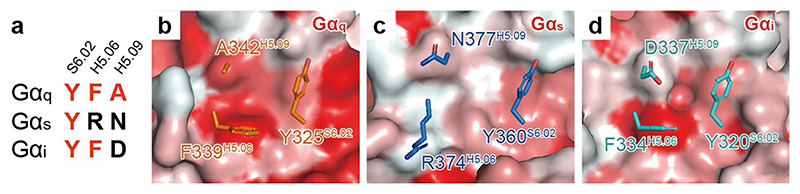
Comparison of the hydrophobic patch in Gα_q_ subunit to the corresponding sites in other G proteins. **a**, Sequence alignment of S6.02, H5.06, and H5.09 from G**α**_q_, G**α**_s_, and G**α**_i_ subunits. Residues at positions S6.02, H5.06, and H5.09 comprise the hydrophobic patch to interact with CCK_A_R ICL3. **b-d**, Surface presentation of the patch by hydrophobicity. Side chains of residues at positions S6.02, H5.06, and H5.09 in G**α**_q_ (**b**), G**α**_s_ (**c**), and G**α**_i_ (**d**) subunits are shown.

**Fig. 5:**
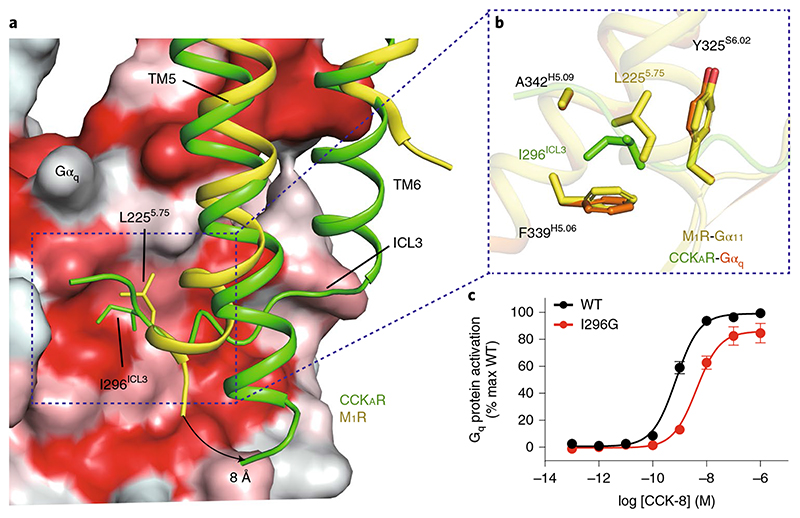
Interaction between the ICL3 loop of CCK_A_R and the Gα_q_ subunit. **a**, I296^ICL3^ of CCK_A_R and L225^5.75^ of M_1_R occupy the same hydrophobic subpocket of the G**α** subunit. The G**α**_q_ subunit is shown as a surface presentation by hydrophobicity (hydrophobic surface in red). An 8-Å outward bend of TM5 of CCK_A_R relative to that of M_1_R is highlighted by a black arrow. **b**, Detail of the interactions between I296^ICL3^(CCK_A_R)/L225^5.75^(M_1_R) and the hydrophobic patch comprising Y325^S6.02^, F339^H5.06^ and A342^H5.09^ of the G**α**_q_ and G**α**_11_ subunits. **c**, BRET assay showing that the I296G mutation decreases the association rate of CCK_A_R with the G_q_ heterotrimer. Data are shown as mean **±** s.e.m. of three independent experiments *(n =* 3), conducted in triplicate.

In the CCK_A_R-G_q_ protein complex structure, CCK_A_R displays a comparable length of TM5 relative to the M_1_R-G_11_ complex^19^. However, the cytoplasmic end of the CCK_A_R TM5 exhibits an 8-Å outward bend (measured at the Cα atoms of A^5.73^), which prevents it from interacting with the Gα_q_ subunit (Fig. 5a). Instead, the ICL3 inserts into the cleft between TM5 of CCK_A_R and the α5-helix of the Gα_q_ subunit (Fig. 5a). Compared to L225^5.75^ in M_1_R, I296^ICL3^ in CCK_A_R interacts with the same hydrophobic patch formed by the side chains of Y325^S6.02^, F339^H5.06^ and A342^H5.09^ in the Gα_q_ subunit, but is buried deeper to create more closely packed hydrophobic contacts (Fig. 5a,b). These hydrophobic interactions are critical to CCK_A_R-G_q_ coupling, as evidenced by our BRET analysis showing that the I296^ICL3^G mutation significantly weakens G_q_ coupling to CCK_A_R but has no impact on G_s_ and G_i_ coupling (Fig. 5c and Supplementary Table 4). This hydrophobic patch, which lies on the outer surface, may be unique for the G_q/11_ subunit. The equivalent residues in the Gα_s_ and Gα_i_ subunits are polar or charged residues, for which it would be energetically unfavorable to form hydrophobic interactions (Extended Data Fig. 7a–d). Indeed, this unconventional ICL3-G_q_ interaction is not seen in the structures of G_s_- and G_i/o_-coupled CCK_A_Rs (Fig. 3b,c). Together, our findings offer structural evidence on the possible role of ICL3 in CCK_A_R-G_q_ coupling preference. Hydrophobic residues on the inner surface of the ICL3 loop of CCK_A_R or the extended TM5 of M_1_R may represent a common feature of G_q/11_-coupled GPCRs.

## Discussion

As the largest family of cell surface receptors, GPCRs have more than 800 members but only couple to four G-protein subtypes. Specific GPCR signaling requires the receptor to couple with either a single or multiple G-protein subtypes^45–47^. Thus, one of the main questions is how does a given GPCR select a G-protein subtype for downstream signal transduction. The critical G-protein determinants of selectivity vary widely for different receptors that couple to specific G proteins. It is thought that G_s_- or G_q_-coupled receptors are relatively promiscuous and to some extent couple to G_i1_ (^22^). However, G_i_-coupled receptors are more selective^22^. The minor outward movement of TM6 contributes to such a superior G_i_ coupling selection in comparison to that of G_s_ (^17,23,44,48,49^). Although proven to be promiscuous, G_q_-coupled receptors tend to adopt an active conformation similar to that of G_i_-coupled GPCRs, reflecting the complexity of the GPCR-G protein coupling mechanism^19,20^. Because CCK_A_R has the ability to couple with different G-protein subtypes, it stands out as a suitable model for studying the promiscuity of G-protein coupling. In this article, we show that TM6 of CCK_A_R undergoes a similar outward displacement relative to G_i/o_-coupled (NTSR1 and M_2_R) and G_q/11_-coupled GPCRs (5-HT_2_AR and M_1_R), but has a smaller shift relative to G_s_-coupled GPCRs (β_2_AR and A_2_AR). CCK_A_Rs share almost identical conformations, whereas G_q_, G_s_ and G_i_ proteins vary in distinct orientations, producing different sizes of receptor-G protein interface. The predominant coupling to G_q_ by CCK_A_R can be explained by its largest interface of the three CCK_A_R-G protein complexes. Structural comparison of the three CCK_A_R-G protein complexes reveals that ‘wavy hook’ residues of the Gα α5-helix and ICL3 of the receptor are important for the coupling promiscuity. In addition, detailed inspections disclose structural clues relative to the recognition mechanism of sulfated CCK-8 by CCK_A_R, in which R197^ECL2^ is a major determinant. Together, our structures provide a framework for better understanding ligand recognition as well as G-protein coupling selectivity and promiscuity by CCK_A_R.

## Methods

### Expression and purification of CCK_A_R-G protein complexes

The WT CCK_A_R (residues 1–428) was used for cryo-EM studies. Full-length CCK_A_R complementary DNA was cloned into a modified pFastBac vector (Invitrogen) containing a hemagglutinin (HA) signal sequence followed by an 8× histidine tag, a double-maltose binding protein tag and a tobacco etch virus (TEV) protease site before the receptor sequence using homologous recombination (using a CloneExpress One Step Cloning Kit, Vazyme; Supplementary Fig. 4a). The N-terminal 1–29 amino acids of Gα_q_ were replaced by the equivalent residues of Gα_π_ to facilitate scFv16 binding^19^. An engineered Gα_s_ construct was generated based on mini-Gα_s_^31^. The N-terminal 1-18 amino acids and the α-helical domain of Gα_s_ were replaced by human Gα_i1_, thus providing binding sites for scFv16 and Fab-G50, respectively^17,19^. Additionally, human Gα_i1_ with two dominant-negative mutations (G203A and A326S^32^) was used to assemble a stable GPCR-G_i_ protein complex. These two cognate mutations also exist in engineered Gα_q_ and Gα_s_ (Supplementary Fig. 4b). Receptor, rat H6-Gβ, bovine Gγ and the specific Gα subunit were co-expressed in *Spodoptera frugiperda* (*sf9*) insect cells (Invitrogen) as previously described^50^. In addition, GST-Ric-8A (a gift from B. Kobilka) was applied to improve the expression of Gα_q_.

ScFv16 was applied to improve the protein stability of CCK_A_R-G_q_ and CCK_A_R-G_i_ complex samples. The monomeric scFv16 was prepared as previously reported^51^. Cell pellets of the co-expression culture were thawed and lysed in 20 mM HEPES, pH 7.4, 100 mM NaCl, 10% glycerol, 5 mM MgCl_2_ and 10 mM CaCl_2_ supplemented with EDTA-free protease inhibitor cocktail (TargetMol). CCK_A_R-G protein complexes were assembled at room temperature (r.t.) for 1 h by the addition of 10 μM CCK-8 (GenScript) and 25 mU ml^-1^ apyrase. The lysate was then solubilized in 0.5% lauryl maltose neopentyl glycol (LMNG), 0.1% cholesteryl hemisuccinate TRIS salt (CHS), and the soluble fraction was purified by nickel affinity chromatography (Ni Smart Beads 6FF, SMART Lifesciences). In the case of CCK_A_R-G_i_ and CCK_A_R-G_q_ complexes, a three-molar excess of scFv16 was added to the protein elute. The mixture was incubated with amylose resin for 2 h at 4 °C. The excess G protein and scFv16 were washed with 20 column volumes of 20 mM HEPES, pH 7.4, 100 mM NaCl, 10% glycerol, 0.01% LMNG, 0.002% CHS and 2 μM CCK-8. TEV protease was then included to remove the N-terminal fusion tags of CCK_A_R. After 1 h of incubation at r.t., the flow-through was collected, concentrated and injected onto a Superdex 200 10/300 column equilibrated in buffer containing 20 mM HEPES, pH 7.4, 100 mM NaCl, 0.00075% LMNG, 0.00025% glycol-diosgenin (GDN), 0.0002% CHS and 10 μM CCK-8. The monomeric complex peak was collected and concentrated to ~5 mg ml^-1^ for cryo-EM studies.

### Cryo-electron microscopy grid preparation and image collection

For preparation of cryo-EM grids, 2.5 μl of each purified CCK_A_R–G protein complex was applied individually onto glow-discharged holey carbon grids (Quantifoil, Au300 R1.2/1.3) in a Vitrobot chamber (FEI Vitrobot Mark IV). The chamber was set to 100% humidity at 4 °C. Extra samples were blotted for 2 s and vitrified by plunging into liquid ethane. Grids were stored in liquid nitrogen for condition screening and data collection usage.

Automatic data collection of CCK-8-CCK_A_R-G protein complexes was performed on an FEI Titan Krios system at 300 kV. The microscope was operated with a nominal magnification of ×81,000 in counting mode, corresponding to a pixel size of 1.045 Å for the micrographs. A total of 5,415 videos for the dataset of the CCK-8–CCK_A_R–G_q_-scFv16 complex, 5,008 for the dataset of the CCK-8–CCK_A_R-G_s_ complex and 4,811 for the dataset of the CCK-8-CCK_A_R-G_i_-scFv16 complex were collected, respectively, by a Gatan K3 Summit direct electron detector with a Gatan energy filter (operated with a slit width of 20 eV; GIF) using SerialEM software. The images were recorded at a dose rate of ~26.7 e Å^−2^ s^−1^ with a defocus ranging from –0.5 to –3.0 μm. The total exposure time was 3 s and intermediate frames were recorded in intervals of 0.083 s, resulting in a total of 36 frames per micrograph.

### Image processing and map reconstruction

Image stacks were subjected to beam-induced motion correction and aligned using MotionCor 2.1. Contrast transfer function (CTF) parameters were estimated by Ctffind4. Data processing was performed using RELION-3.0^52^. Micrographs with measured resolution worse than 4.0 Å and micrographs imaged within the carbon area were discarded, generating 3,806 micrographs for the CCK-8-CCK_A_R-G_q_-scFv16 dataset, 4,963 for the CCK-8-CCK_A_R-G_s_ dataset and 4,543 for the CCK-8-CCK_A_R-G_i_-scFv16 dataset for further data processing. For particle selection, two-dimensional (2D) and 3D classifications were performed on a binned dataset with a pixel size of 2.09 Å. About 2,000 particles were manually selected and subjected to 2D classification. Representative averages were chosen as a template for particle autopicking. The autopicking process produced 3,405,355 particles for the CCK-8-CCK_A_R-G_q_-scFv16 complex, 4,680,972 for the CCK-8-CCK_A_R-G_s_ complex and 4,270,010 for the CCK-8-CCK_A_R-G_i_-scFv16 complex, which were subjected to reference-free 2D classifications to discard bad particles. Initial reference map models for 3D classification were generated by Relion using representative 2D averages. For the CCK-8-CCK_A_R-G_q_-scFv16 complex, the particles selected from 2D classification were subjected to six rounds of 3D classification, resulting in a single well-defined subset with 555,628 particles. For the CCK-8-CCK_A_R-G_s_ complex, particles resulting from 2D classification were subjected to five rounds of 3D classification, resulting in two well-defined subsets with 499,924 particles. For the CCK-8-CCK_A_R-G_i_-scFv16 complex, particles selected from 2D classification were subjected to seven rounds of 3D classifications, resulting in two well-defined subsets with 140,602 particles. Further 3D refinement, CTF refinement, Bayesian polishing and DeepEnhancer processing generated density maps with an indicated global resolution of 2.9 Å for the CCK-8–CCK_A_R–G_q_--scFv16 complex, 3.1 Å for the CCK-8–CCK_A_R-G_s_ complex and 3.2 Å for the CCK-8-CCK_A_R-G_i_-scFv16 complex, respectively, at a Fourier shell correlation of 0.143.

### Model building and refinement

For the CCK_A_R-G_q_ complex, the initial G_q_ protein and scFv16 model were adopted from the cryo-EM structure of the M1R-G11 protein complex (PDB 6OIJ)^19^. The initial CCK_A_R model was generated by an online homology model building tool^53^. All models were docked into the EM density map using Chimera^54^, followed by iterative manual adjustment and rebuilding in COOT^55^ and ISOLDE^56^, and real-space refinement using Phenix programs^57^. The model statistics were validated using Phenix comprehensive validation. A model of the refined CCK_A_R from the CCK_A_R-G_q_ complex was used for the other two complexes. Models from PTH1R-G_s_ (PDB 6NBF) and FPR2-G_i_ (PDB 6OMM) were used as templates for the model building of G_s_ in the CCK_A_R-G_s_ complex and G_i1_-scFv16 in the CCK_A_R-G_i_ complex, respectively. The fitted models were then built in the same way as the CCK_A_R-G_q_ complex. The final refinement statistics are provided in Supplementary Table 1. All figures were prepared using PyMol and Chimera software.

### Radiolabeled ligand-binding assay

The WT or mutant CCK_A_Rs were transiently transfected into HEK 293T/17 cells (purchased from the Cell Bank at the Chinese Academy of Sciences), which were cultured in a poly-D-lysine-coated 96-well plate. After 24 h, the cells were washed twice and incubated with blocking buffer (Dulbecco’s modified Eagle medium (DMEM) supplemented with 33 mM HEPES, and 0.1% (wt/vol) bovine serum albumin (BSA), pH 7.4) for 2 h at 37 °C. After three washes with ice-cold phosphate-buffered saline (PBS), the cells were treated by a constant concentration of ^125^I-CCK-8 (40 pM, PerkinElmer) plus eight different doses of CCK-8 (1 pM to 10 μM) for 3 h at r.t. Cells were washed three times with ice-cold PBS and lysed by 50 μl of lysis buffer (PBS supplemented with 20 mM Tris-HCl and 1% (vol/vol) Triton X-100, pH 7.4). Subsequently, the plates were counted for radioactivity (counts per minute) in a scintillation counter (MicroBeta^2^ plate counter, PerkinElmer) using 150 μl of scintillation cocktail (OptiPhase SuperMix, PerkinElmer).

### G-protein dissociation assay

G-protein dissociation was monitored by BRET experiments performed as previously reported^58^. Briefly, a C-terminal fragment of the G-protein-coupled receptor kinase 3 (GRK3ct) fused to a luciferase serves as a BRET donor. Gβγ dimer is labeled with the fluorescent protein Venus, a BRET acceptor. Upon G-protein heterotrimer activation, free Gβγ-Venus is released and binds to membrane-associated GRK3ct-luciferase, leading to an increased signal detectable by BRET.

HEK 293T/17 cells were seeded onto a 10 μg ml^-1^ Matrigel-coated six-well plate (1 × 10^6^ cells per well). After 4 h of culture, WT or mutant CCK_A_R (0.84 μg), Gα (Gα_q_, Gα_s_ and Gα_i_, 2.1 μg each), Gβ (0.42 μg), Gγ (0.42 μg) and GRK (0.42 μg) were transiently transfected with Lipofectamine LTX reagent (Invitrogen). At 24 h post-transfection, cells were washed once with DMEM medium (no phenol red) and detached by EDTA. Cells were then collected by centrifugation at 1,000 r.p.m. for 5 min and resuspended in DMEM medium. Approximately 75,000 cells per well were distributed in 96-well flat-bottomed white microplates (PerkinElmer). The NanoBRET substrate (furimazine, 25 μl per well, Promega) was added, and the BRET signal (535 nm/475 nm ratio) was determined using an EnVision multilabel plate reader (PerkinElmer). The average baseline value recorded before CCK-8 stimulation was subtracted from BRET signal values.

### NanoBiT G-protein recruitment assay

The recruitment of CCK_A_R to G_i_ protein was detected in *sf*9 cells using the NanoBiT method as previously reported^59^. Briefly, the LgBiT fragment of NanoBiT luciferase was fused to the C terminus of CCK_A_R. SmBiT was fused to the C terminus of the Gβ subunit with a 15-amino-acid flexible linker. CCK_A_R-LgBiT, Gα_i1_, SmBiT-fused human Gβ1 and human Gγ2 were co-expressed in *sf*9 insect cells. Cell pellets were collected by centrifugation after infection for 48 h. The cell suspension was dispensed in a 96-well plate (64,000 cells per well) at a volume of 80 μl diluted in the assay buffer (Hanks’ balanced salt solution buffer supplemented with 10 mM HEPES, pH 7.4) and incubated for 30 min at 37 °C. The cells were then reacted with 10 μl of 50 mM coelenterazine H (Yeasen) for 2 h at r.t. The luminescence signal was measured using an EnVision plate reader (PerkinElmer) at 30-s intervals (25 °C). The baseline was measured before CCK-8 addition for eight intervals, and the measurements continued for 20 intervals following ligand addition. Data were corrected to baseline measurements and the results were analyzed using GraphPad Prism 8.0 (Graphpad Software).

### NanoBiT G-protein dissociation assay

G_s_ activation was measured by a NanoBiT dissociation assay. G-protein NanoBiT split luciferase constructs were generated by fusing the LgBiT in Gα_s_ and the SmBiT to Gγ (a gift from A. Inoue, Tohoku University) as previously reported^60^. In brief, HEK 293T/17 cells were plated in 10-cm plates at a density of 3 × 10^6^ cells per plate. After 24 h, cells were transfected with 1.62 μg receptor plasmids, 0.81 μg Gα_s_-LgBiT, 4.1 μg Gβ and 4.1 μg SmBiT-Gγ using Lipofectamine LTX reagent (Invitrogen). The transiently transfected cells were then seeded into poly-D-lysine-coated 96-well plates (50,000 cells per well) and grown overnight before incubation in assay buffer. Measurement of the luminescence signal was identical to the steps described above.

### Surface expression assay

HEK 293T/17 cells were seeded into a six-well plate and incubated overnight. After transient transfection with WT or mutant plasmids for 24 h, the cells were collected and blocked with 5% BSA in PBS at r.t. for 15 min and incubated with primary anti-Flag antibody (1:300, Sigma-Aldrich) at r.t. for 1 h. The cells were then washed three times with PBS containing 1% BSA followed by 1 h of incubation with donkey anti-mouse Alexa Fluor 488-conjugated secondary antibody (1:1,000, Thermo Fisher) at 4 °C in the dark. After three washes, the cells were resuspended in 200 μl of PBS containing 1% BSA for detection in a NovoCyte flow cytometer (ACEA Biosciences) utilizing laser excitation and emission wavelengths of 488 nm and 519 nm, respectively. For each assay point, ~15,000 cellular events were collected, and the total fluorescence intensity of the positive expression cell population was calculated. The gating strategy and the method for the calculation of expression are shown in Supplementary Fig. 6.

### Molecular docking

Before docking, hydrogens were added to CCK_A_R and the whole system coordinates were optimized with a pH of 7.0. A grid file was then generated on the peptide pocket in our G_q_-coupled CCK_A_R structure. Small-molecule ligands Glaxo-11p, GW-5823 and CE-326597 were prepared in the OPLS3 force field with a pH of 7.0 to generate 3D structures. Finally, glide docking with standard precision was applied to all ligands and the structures with the best docking score were picked as outputs.

### Statistics

All functional study data were analyzed using Prism 8 (GraphPad) and are shown as the mean ± s.e.m. from at least three independent experiments. Concentration-response curves were evaluated with a three-parameter logistic equation. Significance was determined with either a two-tailed Student’s *t*-test or one-way analysis of variance Dunnett multiple comparisons test, and P < 0.05 was considered statistically significant.

## Extended Data

## Supplementary Material

Supplementary material

## Data Availability

Density maps and structure coordinates have been deposited in the Electron Microscopy Data Bank (EMDB) and the Protein Data Bank (PDB) with accession codes EMD-31387 and 7EZH for the CCK-8–CCK_A_R-G_i_-scFv16 complex, EMD-31388 and 7EZK for the CCK-8–CCK_A_R-G_s_ complex, and EMD-31389 and 7EZM for the CCK-8–CCK_A_R-G_q_-scFv16 complex. Source data are provided with this paper.
