# Supplementary material for: Ligand recognition and G-protein coupling selectivity of cholecystokinin A receptor
Source: Nat Chem Biol. Author manuscript; Available in PMC 2021 Dec 1. (PMC8604728; doi:10.1038/s41589-021-00841-3)
Supplement: Supplementary material [file EMS135908-supplement-Supplementary_material.pdf]

nature portfolio

https://doi.org/10.1038/s41589-021-00841-3

Supplementary information

Ligand recognition and G-protein coupling selectivity of cholecystokinin A receptor

In the format provided by the authors and unedited

Supplementary Table 1. **Cryo-EM data collection, refinement, and validation statistics.**

	**CCK_AR/G_q/scFv16** (EMD-31389) (PDB: 7EZM)	**CCK_AR/G_s** (EMD-31388) (PDB: 7EZK)	**CCK_AR/G_i/scFv16** (EMD-31387) (PDB: 7EZH)
Data collection and processing			
Magnification	81,000	81,000	81,000
Voltage (kV)	300	300	300
Electron exposure (e-/ Å2)	80	80	80
Defocus range (μm)	-0.5 ~ -3.0	-0.5 ~ -3.0	-0.5 ~ -3.0
Pixel size (Å)	1.045	1.045	1.045
Symmetry imposed	C1	C1	C1
Initial particle projections (no.)	3, 405, 355	4, 680, 972	4, 270, 010
Final particle projections (no.)	555, 628	499, 924	140, 602
Map resolution (Å)	2.9	3.1	3.2
FSC threshold	0.143	0.143	0.143
Map resolution range (Å)	2.3- 4.3	2.3- 4.3	2.3- 4.3
Refinement			
Initial model used (PDB accession number)	6OIJ	6NBF	6OMM
Model resolution (Å)	3.0	3.2	3.4
FSC threshold	0.5	0.5	0.5
Map sharpening B-factor (Å2)	-97.47	-134.32	-111.38
Model composition			
Non-hydrogen atoms	8999	7196	8860
Protein residues	1170	922	1153
B-factors (Å2)			
Protein	56.03	66.86	63.12
RMSD			
Bond lengths (Å)	0.010	0.010	0.002
Bond angles (°)	1.027	1.010	0.625
Validation			
MolProbity score	1.45	1.39	1.35
Clashscore	4.50	3.85	2.45
Rotamer outliers (%)	0.21	0.26	0.00
Ramachandran Plot			
Favored (%)	96.51	96.57	95.40
Allowed (%)	3.49	3.43	4.60
Disallowed (%)	0.00	0.00	0.00

Supplementary Table 2. **Effects of mutations in the ligand-binding pocket of CCK_AR on CCK-8 binding affinities.**

Radiolabeled ligand ([^{125I}]CCK-8) binding assay was performed to evaluate the ligand-binding affinity of CCK_AR mutants. Binding data are represented mean pKi ± S.E.M. $^{**}P$<0.01, versus wild-type (WT). N.D., not determined. FACS analyses were performed to evaluate the surface expression of the CCK_AR mutants. Expression data are shown as %WT. $^{\dagger}P < 0.05$, $^{\dagger\dagger}P < 0.01$, $^{\dagger\dagger\dagger}P < 0.001$, $^{\dagger\dagger\dagger\dagger}P < 0.0001$, versus WT. All data were analyzed by one-way ANOVA Dunnett multiple comparisons test. No adjustments were made for multiple comparisons.

Mutant	pKi ± S.E.M.	n	*P* value	Expression %	n	*P* value
WT	8.58 ± 0.12	4	1	100	3	1
K105A	7.78 ± 0.22**	3	0.0089	77.86 ± 6.24††	3	0.0020
F107A	N.D.	3	—	71.85 ± 6.84	3	0.0994
T118A	8.73 ± 0.13	3	0.9921	78.06 ± 5.38†	3	0.0153
M121A	8.03 ± 0.15	3	0.1191	74.99 ± 5.48†	3	0.0426
V125A	8.68 ± 0.12	3	0.9993	46.48 ± 1.03††††	3	<0.0001
Y176A	N.D.	3	—	26.63 ± 2.43††††	3	<0.0001
F185A	7.98 ± 0.24	3	0.0742	77.98 ± 4.85	3	0.1029
M195A	8.10 ± 0.26	3	0.2199	85.45 ± 4.52	3	0.5581
C196A	N.D.	3	—	3.24 ± 0.06††††	3	<0.0001
R197A	N.D.	3	—	104.14 ± 5.14	3	0.9993
H210A	8.61 ± 0.12	3	0.9998	81.19 ± 4.32	3	0.2350
I329A	N.D.	3	—	74.22 ± 7.37†	3	0.0334
F330A	8.67 ± 0.09	3	0.9994	27.31 ± 2.74††††	3	<0.0001
A332G	8.43 ± 0.12	3	0.9921	32.50 ± 4.21††††	3	<0.0001
N333A	N.D.	3	—	63.96 ± 3.31†††	3	0.0009
R336A	N.D.	3	—	82.96 ± 5.35	3	0.3489
A343G	N.D.	3	—	50.61 ± 5.37††††	3	<0.0001
E344A	N.D.	3	—	88.00 ± 13.77	3	0.7932
L347A	N.D.	3	—	52.59 ± 1.43††††	3	<0.0001
S348A	N.D.	3	—	98.35 ± 8.18	3	0.9997
I352A	N.D.	3	—	80.35 ± 1.26	3	0.1918
Y360A	8.00 ± 0.09	3	0.0899	82.85 ± 6.85	3	0.3411

Supplementary Table 3. **Coupling activity of CCK_AR with different G proteins.**

BRET assay was performed to evaluate the coupling activity of CCK_AR with different G proteins. Coupling activity data are represented as mean pEC_{50} ± S.E.M. Decreased fold of E_{max} compared to G_q was calculated. BRET experiments were performed in sextuplicate (n=6). Coupling activity data were analyzed by one-way ANOVA Dunnett multiple comparisons test. *P* values, versus Receptor + G_q. Radiolabeled ligand binding assay was used to evaluate the allosteric effects of different G proteins on the binding affinity of CCK-8. The binding affinities are indicated as pKi ± S.E.M. Binding experiments were performed in triplicate (n=3). Binding data were analyzed by one-way ANOVA Dunnett multiple comparisons test. $^*P<0.05$, versus receptor.

**Group**	**G protein-coupling activity of CCK_AR**				**Binding affinity of CCK-8**		
	**pEC_{50} ± S.E.M.**	**n**	***P* value**	**Decreased fold of E_{max}**	**pKi ± S.E.M.**	**n**	***P* value**
Receptor	—	—	—	—	7.92 ± 0.06	3	1
Receptor + G_q	8.42 ± 0.08	6	1	1	$8.28 \pm 0.08^*$	3	0.0143
Receptor + G_i	7.32 ± 0.22	6	0.1230	6.60	7.87 ± 0.07	3	0.9148
Receptor + G_s	7.92 ± 0.65	6	0.5898	20.33	8.02 ± 0.06	3	0.6189

Supplementary Table 4. **Effect of I296G mutation of CCK_AR on G protein-coupling activity.** BRET-based NanoBiT G-protein recruitment and NanoBiT G-protein dissociation assays were performed to evaluate G_q-, G_i-, and G_s-coupling activity, respectively. Data are represented as mean pEC_{50} ± S.E.M. FACS analyses were performed to evaluate the surface expression of CCK_AR mutant. Radiolabeled ligand binding assay was used to evaluate the effects of the mutation on the binding affinity of CCK-8. The binding affinities are indicated as pKi ± S.E.M. All data were analyzed by two-tailed Student's *t*-test by comparing I296G mutants with wild-type (WT) receptor. $^{**}P<0.01$, versus WT. All experiments were performed in triplicate (n=3).

**Mutant**	**G protein-coupling activity**			**Cell Surface expression**	**Binding affinity**
	**pEC_{50} ± S.E.M.**			**Expression %**	**pKi ± S.E.M.**
	**G_q**	**G_i**	**G_s**		
WT	9.14 ± 0.04	6.81 ± 0.12	10.48 ± 0.10	100	8.58 ± 0.12
I296G	8.38 ± 0.09**	6.66 ± 0.08	10.46 ± 0.20	99.52 ± 3.10	8.63 ± 0.13
n	3	3	3	3	3
P value	0.0015	0.3570	0.9330	0.8844	0.7915

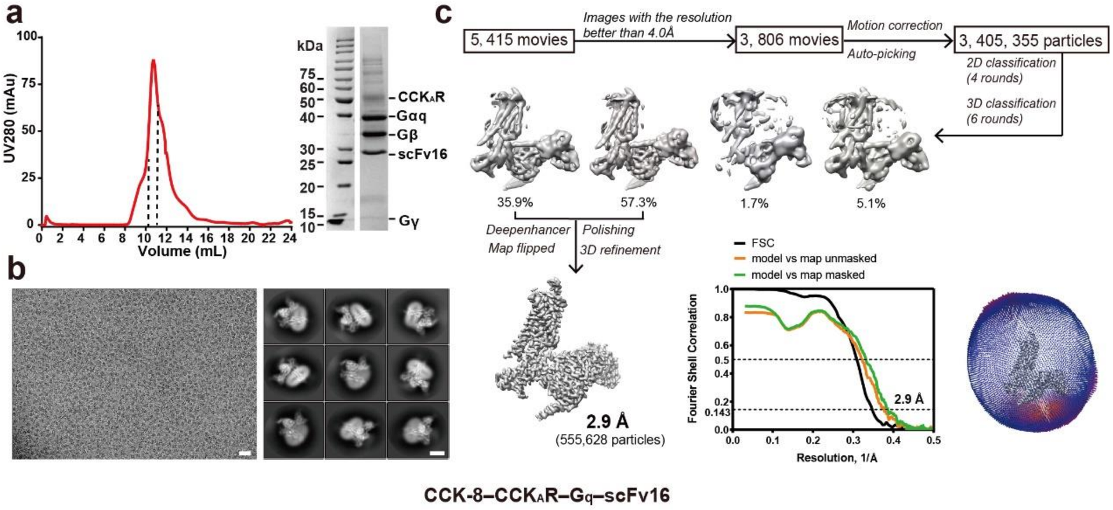

Supplementary Fig. 1│Cryo-EM workflows for structure determination of CCK$_A$R–G$_q$ protein complex. a, Size exclusion chromatography **(**SEC) profile and SDS-PAGE analysis of the CCK-8–CCK$_A$R–G$_q$–scFv16 protein complex sample. **b**, Representative cryo-EM micrograph (scale bar, 30 nm) and 2D classification averages (scale bar, 5 nm) of the CCK-8–CCK$_A$R–G$_q$–scFv16 complex. The data collection was performed once. The 2D averages display different secondary features in different views. **c**, Single-particle cryo-EM data processing flowcharts of the CCK-8–CCK$_A$R–G$_q$–scFv16 by Relion 3.1, including the Euler angle distribution of particles used in the final refinement and the fourier shell correlation (FSC) curves. The global resolution defined at the FSC=0.143 is 2.9 Å.

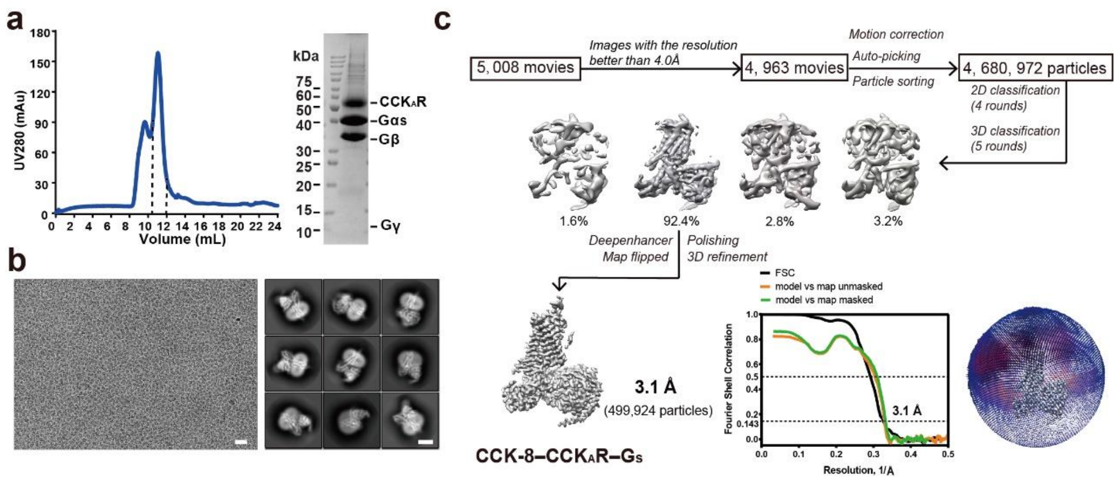

Supplementary Fig. 2 │ Cryo-EM workflows for structure determination of CCK_AR–G_s protein complex.

a, Size exclusion chromatography (SEC) profile and SDS-PAGE analysis of the CCK-8–CCK_AR–G_s protein complex sample. **b**, Representative cryo-EM micrograph (scale bar, 30 nm) and 2D classification averages (scale bar, 5 nm) of the CCK-8–CCK_AR–G_s complex. The data collection was performed once. The 2D averages display different secondary features in different views. **c**, Single-particle cryo-EM data processing flowcharts of the CCK-8–CCK_AR–G_s by Relion 3.0, including the Euler angle distribution of particles used in the final refinement and the fourier shell correlation (FSC) curves. The global resolution defined at the FSC=0.143 is 3.1 Å.

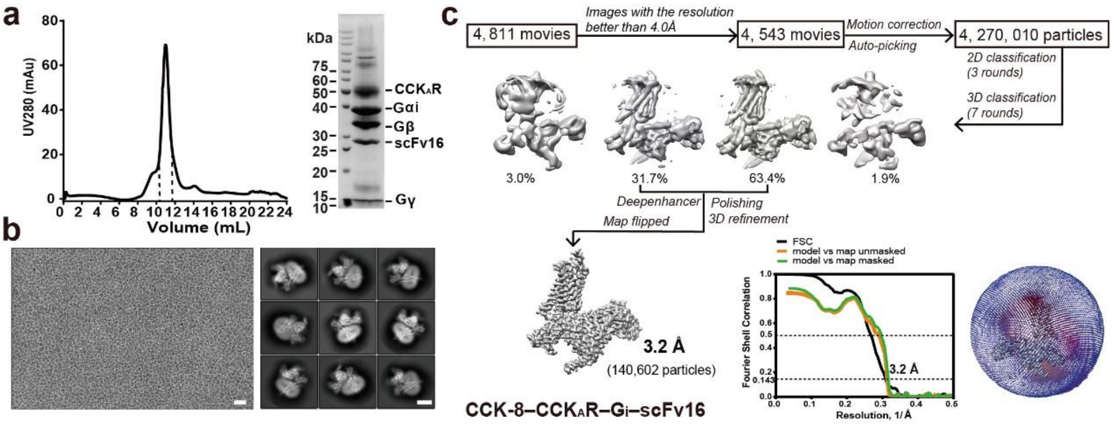

Supplementary Fig. 3│ Cryo-EM workflows for structure determination of CCK_AR–G_i protein complex.

a, Size exclusion chromatography (SEC) profile and SDS-PAGE analysis of the CCK-8–CCK_AR–G_i–scFv16 protein complex sample. **b**, Representative cryo-EM micrograph (scale bar, 30 nm) and 2D classification averages (scale bar, 5 nm) of the CCK-8–CCK_AR–G_i–scFv16 complex. The data collection was performed once. The 2D averages display different secondary features in different views. **c**, Single-particle cryo-EM data processing flowcharts of the CCK-8–CCK_AR–G_i–scFv16 by Relion 3.0, including the Euler angle distribution of particles used in the final refinement and the fourier shell correlation (FSC) curves. The global resolution defined at the FSC=0.143 is 3.2 Å.

a

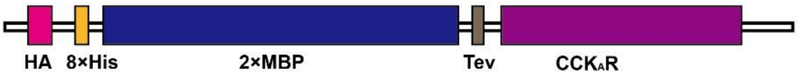

b

Gα$_i$

```
MGCTLSAEDKAAVERSKMIDRNLREDGEKAAREVKLLLLGAGESGKSTIVKQMKIIH
EAGYSEEECKQYKAVVYSNTIQSIIAIIRAMGRLKIDFGDSARADDARQLFVLAGAA
EEGFMTAELAGVIKRLWKDSGVQACFNRSREYQLNDSAAYYLNDLDRIAQPNYIPTQ
QDVLRTRVKTTGIVETHFTFKDLHFKMFDVGAQRSERKKWIHCFEGVTAIIFCVALS
DYDLVLAEDEEMNRMHESMKLFDSICNNKWFTDTSIILFLNKKDLFEEKIKKSPLTI
CYPEYAGSNTYEEAAAYIQCQFEDLNKRKDTKEIYTHFTCSTDTKNVQFVFDAVTDV
IIKNNLKDCGLF
```

Gα$_s$

```
MGCTLSAEDKAAVERSKMIEKQLQKDKQVYRATHRLLLLGADNSGKSTIVKQMRIYH
VNGYSEEECKQYKAVVYSNTIQSIIAIIRAMGRLKIDFGDSARADDARQLFVLAGAA
EEGFMTAELAGVIKRLWKDSGVQACFNRSREYQLNDSAAYYLNDLDRIAQPNYIPTQ
QDVLRTRVKTSGIFETKFQVDKVNFHMFDVGAQRDERRKWIQCFNDVTAIIFVVDSS
DY----------NRLQEALNDFKSIWNNRWLRTISVILFLNKQDLLAEKVLAGKSKI
EDYFPEFARYTTPEDATPEPGEDPRVTRAKYFIRDEFLRISTASGDGRHYCYPHFTC
SVDTENARRIFNDCRDIIQRMHLRQYELL
```

Gα$_q$

```
MGCTLSAEDKAAVERSKMIDRNLREDGEKARRELKLLLLGTGESGKSTFIKQMRIIH
GSGYSDEDKRGFTKLVYQNIFTAMQAMIRAMDTLKIPYKYEHNKAHAQLVREVDVEK
VSAFENPYVDAIKSLWNDPGIQECYDRRREYQLSDSTKYYLNDLDRVADPAYLPTQQ
DVLRVRVPTTGIIEYPFDLQSVIFRMVDVGAQRSERRKWIHCFENVTSIMFLVALSE
YDQVLVESDNENRMEESKALFRTIITYPWFQNSSVILFLNKKDLLEEKIMYSHLVDY
FPEYDGPQRDAQAAREFILKMFVDLNPDSDKIIYSHFTCSTDTENIRFVFAAVKDTI
LQLNLKEYNLV
```

Supplementary Fig. 4│ Receptor and Gα subunits used in the cryo-EM structure determination. a, A schematic illustration of the CCK_AR construct used in cryo-EM studies. HA, hemagglutinin signal sequence; 2×MBP, double-MBP tag. **b**, Protein sequences of Gα$_q$, Gα$_s$, and Gα$_{i1}$ subunits. N-terminal sequence replaced in Gα$_s$ and Gα$_q$ is shown in blue. The two dominant-negative mutations are colored red and underlined. Stabilization mutations derived from the reported mini-Gα$_s$ are highlighted in cyan. AHD domain of the Gα$_s$ is replaced with the equivalent region of Gα$_{i1}$ and colored in gray.

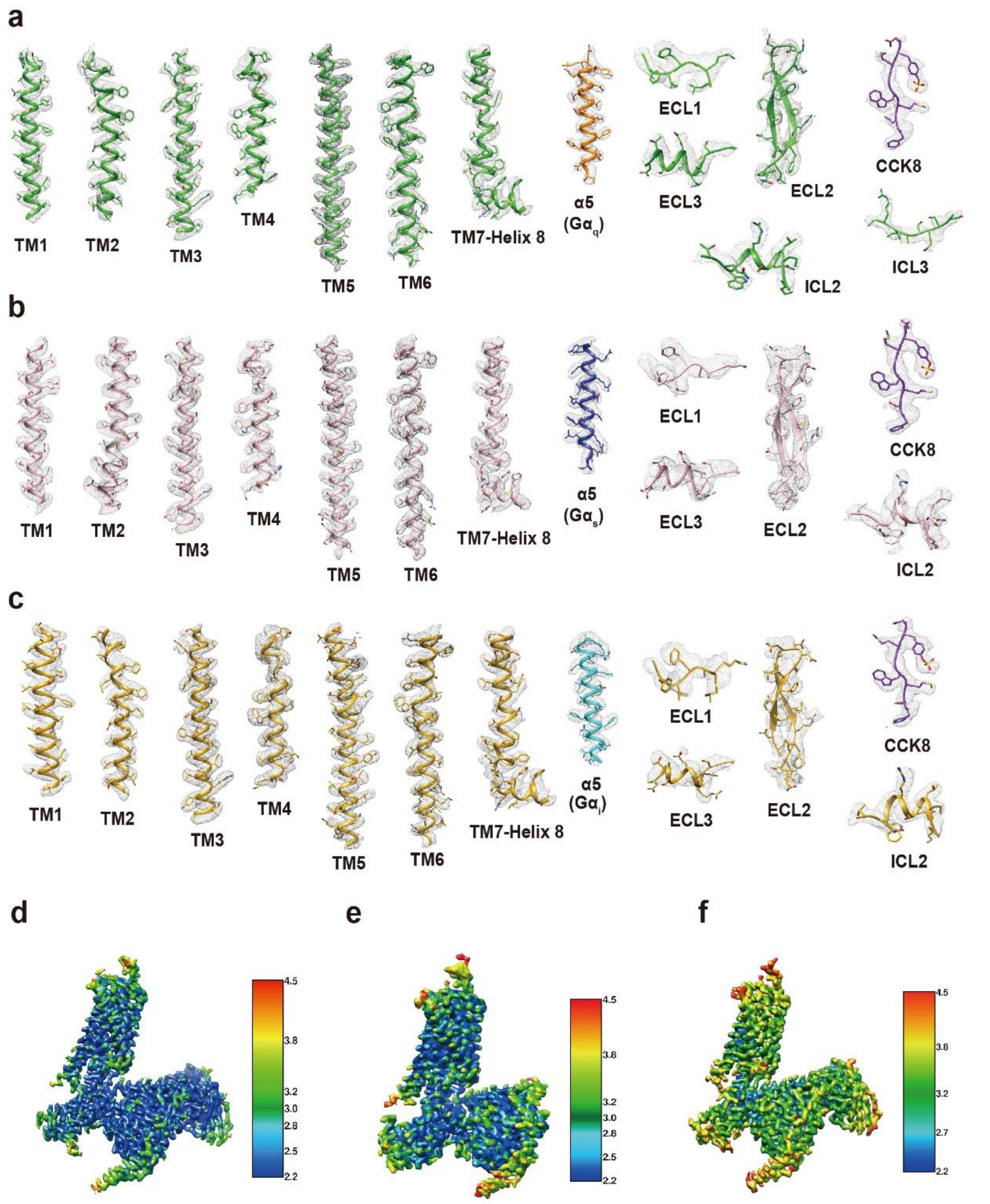

Supplementary Fig. 5│ Local cryo-EM density maps of CCK$_A$R–G protein complexes. a, Cryo-EM density maps of TM1-TM7, ECL1-ECL3, ICL2, ICL3, CCK-8 peptide and α5 helix of Gα$_q$ in the CCK-8–CCK$_A$R–G$_q$–scFv16 structure. **b**, Cryo-EM density maps of TM1-TM7, ECL1-ECL3, ICL2, CCK-8 peptide and α5 helix of Gα$_s$ in the CCK-8–CCK$_A$R–G$_s$ structure. **c**, Cryo-EM density maps of TM1-TM7, ECL1-ECL3, ICL2, CCK-8 peptide and α5 helix of Gα$_i$ in the CCK-8–CCK$_A$R–G$_i$–scFv16 structure. **d-f**, The global density maps of the CCK-8–CCK$_A$R–G$_q$–scFv16 (**d**), CCK-8–CCK$_A$R–G$_s$ (**e**), and CCK-8–CCK$_A$R–G$_i$–scFv16 (**f**) colored by local resolution (Å). The density maps are shown at thresholds of 0.08, 0.055 and 0.05 for the CCK$_A$R–G$_q$, CCK$_A$R–G$_s$ and CCK$_A$R–G$_i$ complex, respectively.

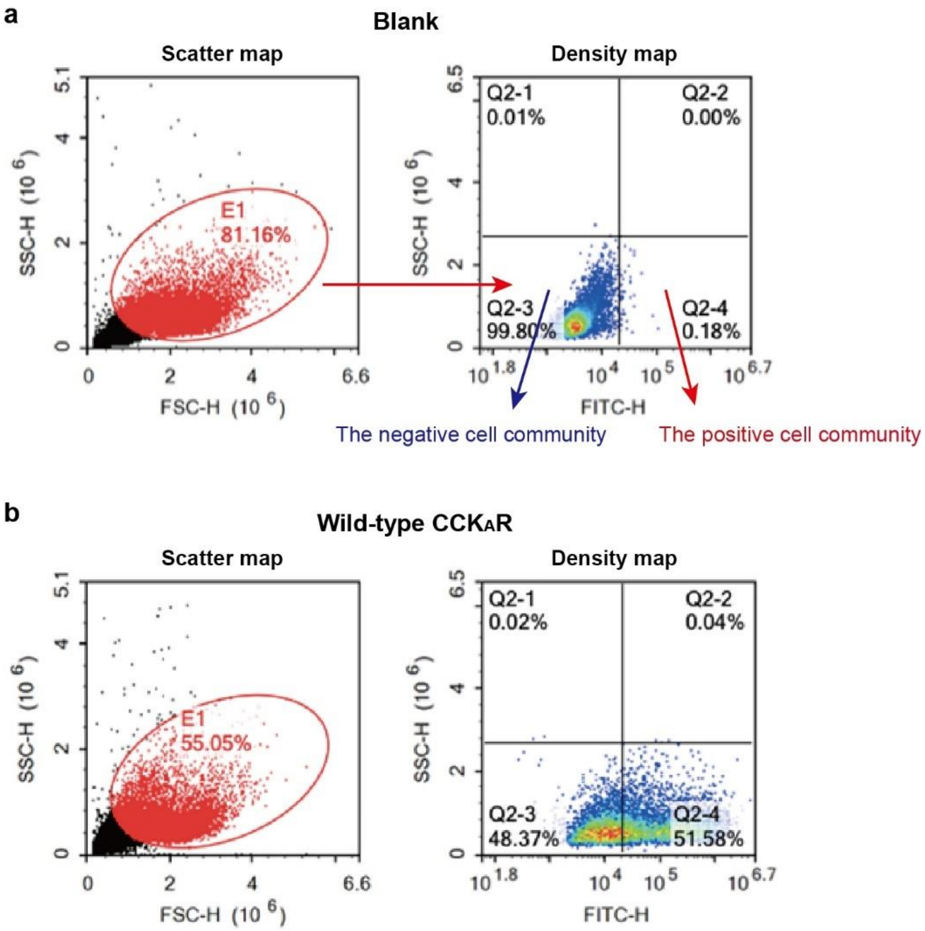

Supplementary Fig. 6 | Gating strategy of cell surface expression assay. Circle a gate E1 in the scatter map (red circle). The cells shown in the density map are all the cells in the gate E1 in the scatter map. Fluorescence signal intensity (FITC) is presented by density map. With the Blank sample as the reference value of background fluorescence signal (**a**), the "quadrant gate" divides the fluorescence signal density map into four quadrants. The third quadrant represents the negative cell community, while the fourth quadrant represents the positive cell community. The expression level of cell surface wild-type (WT) CCK$_A$R (**b**) can be calculated as follows: (M(Q2-4)-M(Q2-3))×(Q2-4% Parent). M, mean fluorescence intensity. The expression level of the CCK$_A$R mutant is calculated similarly to WT CCK$_A$R and then is normalized with the WT to calculate the relative expression value.
